# Event-Related Potential Measures of the Passive Processing of Rapidly and Slowly Presented Auditory Stimuli in MCI

**DOI:** 10.3389/fnagi.2021.659618

**Published:** 2021-04-01

**Authors:** Farooq Kamal, Cassandra Morrison, Kenneth Campbell, Vanessa Taler

**Affiliations:** ^1^School of Psychology, University of Ottawa, Ontario, ON, Canada; ^2^Bruyère Research Institute, Ottawa, ON, Canada

**Keywords:** mild cognitive impairment, MCI, event-related potentials, ERPs, biomarker

## Abstract

Much research effort is currently devoted to the development of a simple, low-cost method to determine early signs of Alzheimer’s disease (AD) pathology. The present study employs a simple paradigm in which event-related potentials (ERPs) were recorded to a single auditory stimulus that was presented rapidly or very slowly while the participant was engaged in a visual task. A multi-channel EEG was recorded in 20 healthy older adults and 20 people with mild cognitive impairment (MCI). In two different conditions, a single 80 dB sound pressure level (SPL) auditory stimulus was presented every 1.5 s (fast condition) or every 12.0 s (slow condition). Participants were instructed to watch a silent video and ignore the auditory stimuli. Auditory processing thus occurred passively. When the auditory stimuli were presented rapidly (every 1.5 s), N1 and P2 amplitudes did not differ between the two groups. When the stimuli were presented very slowly, the amplitude of N1 and P2 increased in both groups and their latencies were prolonged. The amplitude of N1 did not significantly differ between the two groups. However, the subsequent positivity was reduced in people with MCI compared to healthy older adults. This late positivity in the slow condition may reflect a delayed P2 or a summation of a composite P2 + P3a. In people with MCI, the priority of processing may not be switched from the visual task to the potentially much more relevant auditory input. ERPs offer promise as a means to identify the pathology underlying cognitive impairment associated with MCI.

## Introduction

Mild cognitive impairment (MCI) is a condition in which individuals demonstrate cognitive impairment with no impairments in social or occupational function. MCI may represent a transitional stage between healthy aging and Alzheimer’s disease (AD) with 20–40% of people with MCI progressing to dementia (Roberts and Knopman, 2013). The early identification of MCI and prediction of decline associated with progression to AD has been the subject of intense research (Sperling et al., 2011). Much of this research is devoted to the development of a simple, low-cost, and readily available biomarker to determine the early signs of neuropathology underlying AD.

Neuropsychological tests are often used to diagnose AD. Performance on almost all neuropsychological and cognitive tasks will inevitably be affected by the participant’s ability and willingness to maintain attention (Sturm et al., 1999; Buschman and Miller, 2010; Oberauer, 2019). Attentional control and the maintenance of attention may be a challenge for older adults, and particularly for people with MCI (Saunders and Summers, 2010). A good deal of early processing of sensory input is said to be automatic; that is, it is completed whether or not the participant attends to the sensory channel. Determining the extent of processing of unattended input is methodologically difficult. The processing of unattended input can be measured by event-related potentials (ERPs), the changes in the electrical activity of the brain elicited by an external stimulus or internal psychological event. ERPs consist of a series of negative- and positive-going components, thought to reflect different aspects of information processing. Some of these ERP components are elicited independently of attention.

All auditory stimuli elicit an obligatory negative component, the N1, occurring around 100 ms post-stimulus, followed by a later positivity, the P2, occurring around 180 ms. In the classic Näätänen (1990) model of auditory processing, a transient detector system detects abrupt onsets and offsets of auditory stimuli. The output of this system, reflected by the amplitude of N1, varies directly with the rate of stimulus presentation and the energy (intensity) of the stimulus, thus defining its salience. N1 and the P2 will therefore be larger for higher intensity auditory stimuli and stimuli presented slowly. Critically, it has long been known that attention to the auditory channel has relatively little effect on N1 and P2 (Picton and Hillyard, 1974), especially when stimuli are presented slower than every 1 s (Schwent et al., 1976; Hansen and Hillyard, 1988; Woldorff, 1995).

In the Näätänen (1990) model, sufficiently high activation of the transient detector system will result in an interrupt signal being sent to the frontoparietal network controlling processing priorities (Goulden et al., 2014). Attention may then be switched from the ongoing cognitive activities to the processing of the highly salient stimulus event. A later positivity, the P3a, peaking between 200 and 300 ms, is thought to reflect processes associated with the switching of attention (Escera et al., 1998; Masson and Bidet-Caulet, 2019).

The P3a is often elicited in oddball paradigms by a deviant representing a large change from the frequently occurring standard stimulus. There is evidence that a P3a can also be elicited by a single, rarely presented stimulus. When the rate of stimulus presentation is very slow (> than every 10 s), N1 and P2 become very large and their peak latencies are delayed by about 20–30 ms (Alcaini et al., 1994; Budd et al., 1998; Muller-Gass et al., 2008; Pereira et al., 2014). Berti (2013) questioned whether this P2 might be better described as a P3a. The Berti study required subjects to decide on a visual stimulus. On 13% of trials, the visual stimulus was preceded by an irrelevant auditory stimulus. Performance on the visual task subsequently deteriorated, compared to trials in which no auditory stimulus preceded the visual stimulus. This suggested that attention had been switched from the processing of the visual task to the processing of the auditory stimulus. Such processing is associated with the P3a rather than the P2. In the present study, we describe the positivity following the very slow presentation of the stimulus as a composite P2/P3a. Rinne et al. (2006) and Muller-Gass et al. (2007) employed oddball paradigms in which the rare deviant was created by either decreasing or increasing the intensity of the standard. Only the intensity increase elicited a large P3a, presumably because it resulted in large output from the transient detector system. In this regard, Cecchi et al. (2015) employed an oddball paradigm with a white noise burst deviant. The intensity of white noise at times increases and as such will be detected by the transient detector system. A P3a was elicited by the noise burst in healthy older adults but was reduced in amplitude in people with MCI.

There is disagreement about how the N1 and P2 change with aging. In most studies, stimuli are presented relatively rapidly, every 1–3 s. Many of these studies have failed to find N1 or P2 differences between younger and older adults, while some have reported larger responses for younger adults and others have reported larger responses for older adults (Pfefferbaum et al., 1980; Cranford and Martin, 1991; Bertoli et al., 2005; Harkrider et al., 2005; Čeponiene et al., 2008; McCullagh and Shinn, 2013; Stothart and Kazanina, 2016; Kamal et al., 2021). Stimulus features and experimental parameters differ widely across studies, making comparison difficult. In general, even when differences between younger and older participants are observed, they tend to be small. More consistent results have been observed when stimuli are presented very slowly. Berti et al. (2017) and Kamal et al. (2021) varied the rate of stimulus presentation of the to-be-ignored auditory stimuli. When the auditory stimuli were presented very slowly (every 10 and 12 s respectively), the amplitude of both N1 and P2/P3a was much reduced in the older compared to younger adults.

A limited number of studies have examined the N1 and P2 in people with MCI (for a review see Morrison et al., 2018). When the stimuli are presented relatively rapidly, most studies have not found N1 and P2 differences between healthy older adults and people with MCI (Golob et al., 2002; Lai et al., 2010; Lister et al., 2016; Bidelman et al., 2017; Buján et al., 2019). Some studies have reported a somewhat larger N1 or a reduced P2 in people with MCI, at least in certain conditions (Irimajiri et al., 2005; Golob et al., 2007; Lister et al., 2016; Buján et al., 2019). While some of these differences have been attributed to the severity of MCI, experimental parameters again tend to vary widely across studies.

The effects of very slowly presented stimuli have yet to be examined in people with MCI. The large age-related changes in N1 and P2/P3a elicited by auditory stimuli presented very slowly offer much promise for early identification of MCI. The paradigm used by Kamal et al. (2021) has many advantages. Testing can be completed within 15 minutes. Moreover, the participant does not need to attend to the auditory stimuli; the ERPs are elicited passively, independent of attention. In the present study, participants were asked to ignore the auditory stimuli while engaged in a visual task. We compared the passive processing of the auditory stimuli in people with MCI and cognitively healthy older adults. The auditory stimuli were presented rapidly and very slowly in separate conditions. Berti et al. (2017) and Kamal et al. (2021) observed large N1 and P2/P3a differences between younger and older adults only when stimuli were presented very slowly. It was therefore expected that with the additional decline observed in people with MCI compared to healthy older adults, ERP amplitude differences would also only be observed in the slow condition.

## Materials and Methods

### Participants

Forty-one participants took part in this study. One participant was excluded from the analysis because of noisy EEG data (see “EEG Data Recording” section). A total of 40 participants’ data were therefore analyzed: 20 cognitively healthy older adults (12 females; age range = 67–81 years; *M* = 72.4 years) and 20 people with MCI (10 females; age range = 68–84 years; *M* = 74.2 years). Older adults were recruited through word-of-mouth and announcements displayed at community centers. Participants with MCI were recruited from the Bruyère Memory Program. They were diagnosed with MCI based on the clinical history and a neurological exam by a physician with expertise in neurodegenerative conditions. They underwent a CT scan and blood work to rule out reversible causes of cognitive impairment. Participants were not included if their cognitive decline was thought to be related to other comorbidities.

The Montreal Cognitive Assessment (MoCA) was used to screen for cognitive decline (Nasreddine et al., 2005). The cutoff for the MoCA in cognitively healthy older adults was 24. Healthy older adults scored significantly higher (*p* < *0*.001) on the MoCA (*M* = 27.05, *SD* = 1.46) than people with MCI (*M* = 22.79, *SD* = 3.24). Participants also completed a one-hour neuropsychological battery to assess general cognitive functioning (see Supplementary Table 1). The healthy older adults also participated in the Kamal et al. (2021) study. All participants reported no history of neurological or psychiatric conditions. All participants reported normal hearing but also completed pure tone audiometric testing for 500, 1,000, and 2,000 Hz frequencies.

This study was approved by the University of Ottawa and Bruyère Research Institute ethics boards. Participants provided informed written consent before starting the study and an honorarium was given as compensation.

### Stimuli and Procedure

Participants were seated in a sound-attenuated testing room. A single 80 dB SPL (sound pressure level) 1,000 Hz pure tone auditory stimulus, having a total duration of 55 ms (5 ms rise/fall time) was presented binaurally through Sony MDR-V6 headphones. The stimuli were presented every 1.5 s in a fast condition and every 12.0 s in a slow condition. A total of 400 and 50 stimuli were presented in the fast and slow conditions, respectively. Each condition lasted 10 min. The order of the two conditions was randomized across participants. The auditory conditions were presented a second time in reverse order. A total of 100 and 800 stimuli were therefore presented in the slow and fast conditions, respectively. The repetition of stimulus presentations served to reduce the amplitude of the random background noise in the EEG.

Participants were instructed to watch a silent, subtitled video and to ignore the presentation of the irrelevant auditory stimuli. Processing of the auditory stimuli thus occurred passively.

### EEG Data Recording

Continuous EEG activity was recorded from 31 active silver-silver chloride electrodes, attached to an electrode cap placed according to the international 10-10 system. An EOG electrode was placed on the infraorbital ridge of the left eye to monitor vertical eye movements and blinks. An electrode placed on the tip of the nose served as a reference for all channels. An advantage of active electrodes is that impedance can be relatively high (Kappenman and Luck, 2010). Inter-electrode impedance was kept below 20 kΩ. The impedance at F3, Fz, F4, and C3, Cz, C4, which comprised regions of interest (ROIs), was below 10 kΩ. The EEG and EOG signals were sampled at a rate of 500 Hz.

The EEG was then visually examined to remove channels containing high levels of noise. These channels were substituted by interpolating the data of the surrounding electrode sites (Perrin et al., 1989). Interpolation was not applied to any of the ROI sites. The data of one participant were removed from further analysis because more than four channels with excessive noise were rejected. A 0.5 Hz high-pass and a 20 Hz low-pass digital filter (24 dB/octave roll-off) were then applied to the data.

Eye movement and blink artifacts occurring independently of EEG activity were identified and corrected using Independent Component Analysis (ICA; Makeig et al., 1996). To do so required computation of vertical and horizontal EOG activity. A vertical EOG was computed by subtracting FP1 from the inferior orbital activity. Horizontal eye movements were computed by subtracting the FT9 and FT10 activity. The EEG was subsequently reconstructed into single 700 ms epochs starting 100 ms before stimulus onset. The average of all activity in the pre-stimulus period served as a zero-voltage baseline. Drifts in post-stimulus voltage from this baseline were then corrected for each epoch. Epochs containing EEG activity exceeding ±100 μV were subsequently rejected from the averaging. In the fast condition, fewer than 1% of trials were rejected for healthy older adults, while fewer than 3% were rejected for people with MCI (*p* = 0.20). In the slow condition, fewer than 2% of trials were rejected for either group.

### ERP Quantification

The amplitude of N1 and P2/P3a was quantified as the mean of all data points within ± 25 ms of their peak amplitude identified in the grand average of each group. In both groups, N1 peaked at 95 ms in the fast condition and 115 ms in the slow condition. The subsequent P2/P3a positivity peaked at 205 ms in the fast condition and 230 ms in the slow condition.

N1 and P2/P3a were quantified at frontal (F3, Fz, F4) and central (C3, Cz, C4) ROIs, where they are largest. Separate 2-way ANOVAs were initially run at these ROIs for both the N1 and P2/P3a with a single between-subjects factor, Group (Older, MCI), and a single within-subjects factor, Rate of Presentation (Fast, Slow). The results were quite similar at both electrode sites. For this reason, the data were collapsed across ROIs. A 3-way ANOVA was then run with the between-subjects factor, Group (Older, MCI), and two within-subjects factors, Rate (Fast, Slow) and ROI (Frontal, Central). Previous research has shown large ERP differences between younger and older participants but only when stimuli were presented very slowly. We, therefore, expected to observe differences between MCI and healthy older adults only in this condition. For this reason, planned comparisons were run on interactions involving Group and Rate of Presentation.

## Results

**Figure 1:**
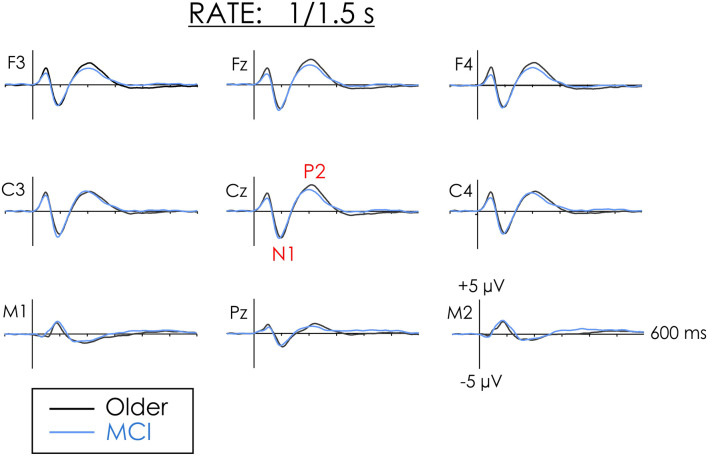
Grand averaged event-related potentials (ERPs) from healthy older adults and people with mild cognitive impairment (MCI) in the fast rate of presentation condition. N1 and P2 amplitude did not differ between the two groups.

**Figure 2:**
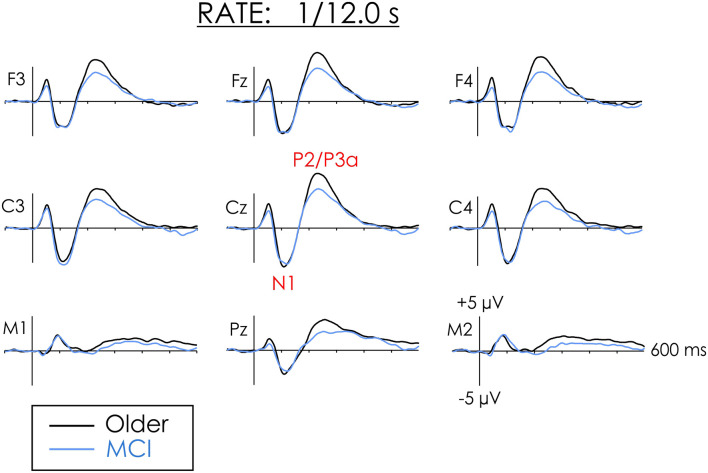
Grand averaged ERPs from healthy older adults and people with MCI in the slow rate of presentation condition. The amplitude of N1 did not differ between the groups. The amplitude of P2 was larger for healthy older adults than people with MCI at both the frontal and central regions of interest (ROIs).

Figures 1, 2 illustrate the multi-channel ERPs for both groups in the fast and slow conditions, respectively. As may be observed, a robust negative peak, N1, occurring at about 100 ms was elicited in both conditions followed by a later positivity, P2/P3a, occurring at about 200 in the fast condition and 230 ms in the slow condition.

### N1

A main effect of Rate of Presentation was observed for the amplitude of N1, *F*_(1,38)_ = 29.05, *p* < 0.001, $\eta_{\text{p}}^{2}$ = 0.43. N1 was larger in the slow than in the fast condition. Overall Group differences were not significant (*F* < 1) and interactions involving Group were not significant (*F* < 1). Thus, regardless of the rate of presentation, the amplitude of N1 did not significantly differ between groups at either frontal or central ROIs.

**Figure 3:**
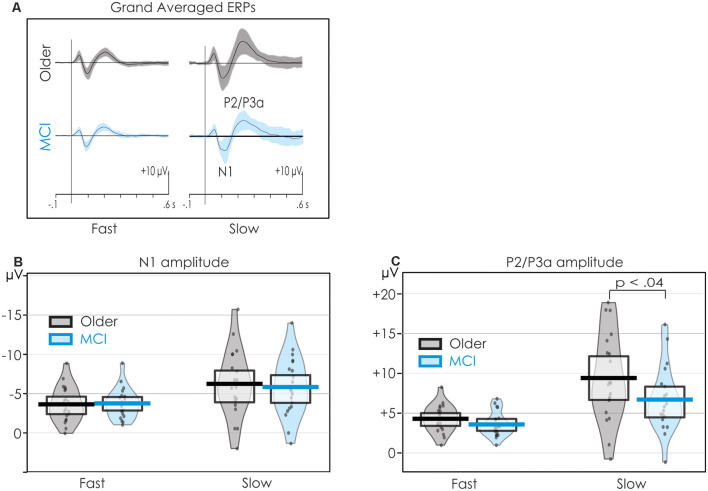
Pirateplots of N1 and P2 data providing both descriptive and inferential statistics. Data are collapsed across all central electrode (C3, Cz, C4) sites. The grand averages and SDs (shaded) are illustrated in panel **(A)**. The mean amplitudes of N1 and P2 (thick, solid horizontal line), 95% confidence intervals (CIs; light horizontal box), smooth frequency distribution (shaded area), and individual data points (jittered) are presented in panels **(B,C)** respectively. The mean amplitude of N1 did not differ between the groups in the fast condition. On the other hand, P2 was larger for the healthy older adults when stimuli were presented slowly.

Figure 3A presents the grand averaged ERPs at Cz including SDs around the average. A pirate plot illustrating both descriptive and inferential statistics (Phillips, 2017) of the N1 data is presented in Figure 3B. As may be observed, the confidence intervals (CIs) for N1 were very similar for both groups. There was considerable overlap between healthy older adults and people with MCI in both the fast and slow conditions.

### P2/P3a

An overall significant main effect of the Rate of the presentation was also observed for P2/P3a. P2/P3a was larger in the slow than the fast condition, *F*_(1,38)_ = 28.30, *p* < 0.001, $\eta_{\text{p}}^{2}$ = 0.43. The Group × Rate interaction was not significant *F*_(1,38)_ = 2.53, *p* = 0.12, $\eta_{\text{p}}^{2}$ = 0.06. The trend of the interactions was, however, in keeping with *a priori* predictions. Follow-up Fisher’s Least Square Significance procedures revealed the source of the interactions. Group differences were not significant in the fast condition (*p* < 0.20). However, in the slow condition, P2 was significantly larger for healthy older adults than for people with MCI (*p* < 0.03). The Group × Condition × ROI interaction was not significant, *F* < 1.

A pirate plot of the P2/P3a at the central ROI is presented in Figure 3C. The mean amplitude of the P2/P3a was larger for the older than the MCI group, but only in the slow condition. There was, however, considerable overlap in individual participants’ amplitudes within the two groups.

### Changes Across Quarters

**Figure 4:**
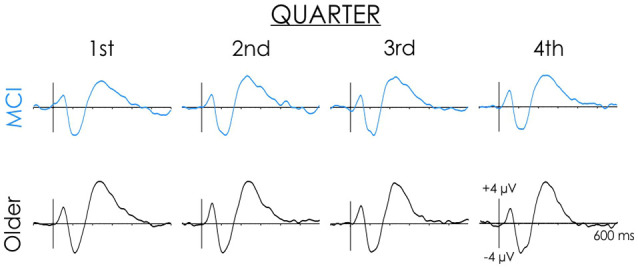
Healthy older adult and MCI grand averaged ERP waveforms across the four quarters of the slow rate of presentation condition. Data presented are from the Cz electrode site. Note the N1 and P2 did not change across the four quarters for either healthy older adults or people with MCI.

A reduced P2/P3a was observed in people with MCI in slow condition (see Figure 4). Possibly, their ERPs varied over time, while being more consistent for healthy older adults. The data were separated into four equal quarters to explore changes over time. The first trial was excluded from this analysis because it marked the initiation of a new condition. Thus, for the slow condition trials 2–13, 14–25, 26–37, and 38–49 were averaged separately. The main effect of the Quarter was not significant (*F* < 1). Importantly, the Group x Quarter interaction was also not significant (*F* < 1).

### Correlations

Correlations were also computed between the P2/P3a amplitude and the MoCA scores within the MCI group. In the fast condition, no significant correlations were found *r* = 0.24, *p* = 0.22 at Fz, and *r* = 0.32, *p* = 0.22 at Cz. In the slow condition, the correlations were also not significant, *r* = 0.32, *p* = 0.19 at Fz, and *r* = 0.40, *p* = 0.09 at Cz.

### Scalp Distribution

N1 and P2/P3a were both large over fronto-central areas of the scalp. Interactions involving Site and Group and Site and Rate were not significant for either N1 or P2/P3a (*F* < 1 in all comparisons). Spline-interpolated scalp distribution maps of N1 are illustrated in the Supplementary Figure 1.

## Discussion

The auditory stimulus in both the fast and slow conditions elicited a robust N1 and P2/P3a. In both healthy older adults and people with MCI, the amplitude of N1 and P2/P3a increased and their latencies were prolonged when the stimuli were presented very slowly. This finding replicates several other studies in younger adults (Alcaini et al., 1994; Budd et al., 1998; Muller-Gass et al., 2008; Pereira et al., 2014). There is evidence that the sources of the auditory N1 differ depending on the rate of stimulus presentation. When stimuli are presented relatively rapidly, the sources have been identified to be in and around the auditory cortex. When stimuli are presented very slowly, the large increase in the amplitude of the N1 and P2/P3a, and their prolonged latencies has been explained by activation of additional widespread sources, particularly in the frontal lobes (Sams et al., 1993; Alcaini et al., 1994; Giard et al., 1994; McEvoy et al., 1997). Many imaging studies have noted a deterioration and loss of function in the frontal regions with age and in early dementia (Driscoll et al., 2009; Machulda et al., 2009; Madden et al., 2012; Salami et al., 2012).

It was expected that differences between healthy older adults and people with MCI would be largest when stimuli were presented very slowly, and smallest when stimuli were presented rapidly. The finding that the N1 and P2 amplitudes did not differ between the two groups in the fast condition is consistent with other studies (Golob et al., 2002; Lai et al., 2010; Lister et al., 2016; Bidelman et al., 2017; Buján et al., 2019). However, contrary to expectations, the amplitude of N1 was not significantly reduced in people with MCI when stimuli were presented slowly. The amplitude of N1 can be used to define the salience of stimulus input. N1 amplitude is larger when transient energy (intensity) is higher or when the time between the onset of stimuli is very long (i.e., when stimuli are presented slowly). In the present study, there is thus little evidence that at the early level of processing, people with MCI have a deficit in computing the salience of unattended auditory stimuli compared to healthy older adults.

In the present study, when stimuli were presented very slowly, planned comparisons indicated that the P2/P3a was significantly larger in older adults than in people with MCI. Its peak latency, around 230 ms, is more consistent with that of a P3a than a P2. Distinguishing between the P2 and P3a processes can be difficult because they may overlap and summate both temporally (occurring at about the same time) and spatially (sharing a similar scalp distribution).

When stimuli were presented slowly, it could be argued that the reduced P2/P3a in people with MCI is a result of a variable response within this condition. The reduction in this positivity in people with MCI in the slow condition might have been a result of rapid habituation, or a fatigue effect throughout the study. Ruusuvirta (2021) notes that the ERP response will be large in the initial trials but will decay upon repetition of the stimulus. Thus, in people with MCI, it is possible that the P2/P3a response was large in the initial trials but subsequently became much smaller. By contrast, in healthy older adults, the P2/P3a response may not have varied within the condition. However, there was little evidence to support this notion. When the averages were computed across each quarter of the study, ERP response showed little variance over time for either group.

The reduced P2/P3a in MCI, also observed by Cecchi et al. (2015), supports the view that the operations of the frontoparietal network may be dysfunctional in MCI. At first glance, the reduced P2/P3a does seem to contradict the theory that people with MCI are less able to inhibit the processing of irrelevant, unattended stimulus input (Belleville et al., 2008; Johns et al., 2012; Rabi et al., 2020; but see Rey-Mermet and Gade, 2018). In the present study, although the auditory stimuli were not attended to, their very rare occurrence should have been deemed to be a potentially highly *relevant* event. The amplitude of N1 did not significantly differ between people with MCI and healthy older adults. This finding suggests that people with MCI can establish the relevance/salience of the incoming stimulus event. To determine the actual relevance of such input would require a switch of processing priorities and the continuation, rather than the inhibition, of further processing. The dysfunction in people with MCI, therefore, appears to occur later as a result of a reduction in the ability to determine processing priorities.

In people with MCI, the correlational analyses indicated a small positive relationship between the amplitude of P2/P3a and MoCA scores. Nevertheless, the correlations did not attain statistical significance, perhaps because of the limited range of MoCA scores. It is also possible that the specific cognitive functions reflected by the P2/P3a are different from the more global cognitive functions measured by the MoCA.

### Conclusion

Several studies have proposed the use of electrophysiological measures as a biomarker of MCI (Gu and Zhang, 2017; Morrison et al., 2018; Paitel et al., 2020). The very simple paradigm used in the present study has the advantage that it could be readily implemented on almost any low-cost commercial system. Testing can be completed in a short 15-min period. It has the marked advantage that the ERP responses elicited by the slowly-presented auditory stimuli occur relatively independent of attention, task demands, and what the participant is doing. From a clinical and applied perspective, whether the positivity occurring around 230 ms reflects P2 or P3a activity may be somewhat incidental. What is critical for its use as a biomarker is how accurately ERPs can classify people with MCI and cognitively healthy older adults. Despite overlap among individual participants, the P2/P3a group differences in the slow condition were significant. ERPs thus offers promise as a means to identify the pathology underlying cognitive impairment associated with MCI. Future research should examine the effects of even slower rates of stimulus presentation and different intensity levels. The use of an oddball paradigm which includes white noise and novel environmental sound deviants, known to elicit a large P3a, might also be employed. A longitudinal design should also be employed to examine differences between MCI participants who convert to dementia and those who remain stable. These studies could reveal the electrophysiological changes associated with conversion to dementia at an individual level.

## Data Availability Statement

The datasets presented in this article are not readily available because research ethics board restrictions preclude the sharing of data. Requests to access the datasets should be directed to vtaler@uottawa.ca.

## Ethics Statement

The studies involving human participants were reviewed and approved by the University of Ottawa and Bruyère Research Institute Ethics Boards. The patients/participants provided their written informed consent to participate in this study.

## Author Contributions

FK, CM, KC, and VT contributed to the rationale and the design of the study and edited and approved the final manuscript. The manuscript was written by FK. FK and CM assisted with the collection and analysis of the EEG data. KC, CM, and VT provided feedback and revisions on written drafts of the manuscript. All authors contributed to the article and approved the submitted version.

## Conflict of Interest

The authors declare that the research was conducted in the absence of any commercial or financial relationships that could be construed as a potential conflict of interest.
