# Supplementary material for: Event-Related Potential Measures of the Passive Processing of Rapidly and Slowly Presented Auditory Stimuli in MCI
Source: Front Aging Neurosci. 2021 Apr 1;13:659618. doi: 10.3389/fnagi.2021.659618 (PMC8046914; doi:10.3389/fnagi.2021.659618)
Supplement: Supplementary file 1 [file Table_1.DOCX]

**Supplementary Table 1:** Healthy older adult and MCI means (SD) for demographic neuropsychological measures.

|  | Healthy Older Adult | MCI | *p* |
| --- | --- | --- | --- |
| Age | 72.41(3.85) | 74.21(4.69) | .12 |
| Education | 16.00(2.85) | 16.61(3.57) | .56 |
| MoCA | 27.05(1.46) | 22.79(3.24) | **<.001** |
| Digit Span Forward | 10.85(2.39) | 10.16(2.17) | .18 |
| Digit Span Backward | 7.80(2.42) | 7.47(2.14) | .33 |
| Letter # Sequencing | 10.45(2.21) | 8.95(2.01) | **.017** |
| WCST | 3.75(1.33) | 2.50(1.33) | **<.001** |
| Stroop1-3 | 56.75(22.84) | 60.39(12.40) | .28 |
| Stroop2-3 | 29.85(13.25) | 30.28(10.03) | .46 |
| Digit Symbol-Written | 44.95(5.47) | 34.37(11.58) | **<.001** |
| Digit Symbol-Oral | 52.40(9.32) | 40.32(11.77) | **<.001** |
| FAS Fluency | 42.00(9.70) | 34.94(7.70) | **.008** |
| Animals | 20.45(5.36) | 13.67(4.80) | **<.001** |

*Notes:* Independent sample one tailed t-test were computed on the neuropsychological scores as it was expected that MCI would score lower than healthy older adults. WCST= Wisconsin Card Sorting Test (categories completed). Stroop1-3= a subtraction of Stroop 3 which requires participants to name the ink color of color words printed in a different color (e.g., the word “RED” printed in green ink) from Stroop1 which requires participants to read name of colors. Stroop 2-3 = a subtraction of Stroop3 from Stroop 2 which requires participants to name the color of “X’s”. Digit Symbol-Written = match the digit to corresponding symbol by writing the answer. Digit Symbol-Oral= match the digit to corresponding symbol by reading the answer aloud. FAS Fluency = is a sum score of three individual fluency tasks in which participants are asked to list as many words as they can that begin with three letters (F, A, and S) in 1 minute. Animal fluency = participants are asked to list as many animals as they can in 1 minute.
