# Supplementary figures and images for: Event-Related Potential Measures of the Passive Processing of Rapidly and Slowly Presented Auditory Stimuli in MCI
Source: Front Aging Neurosci. 2021 Apr 1;13:659618. doi: 10.3389/fnagi.2021.659618 (PMC8046914; doi:10.3389/fnagi.2021.659618)

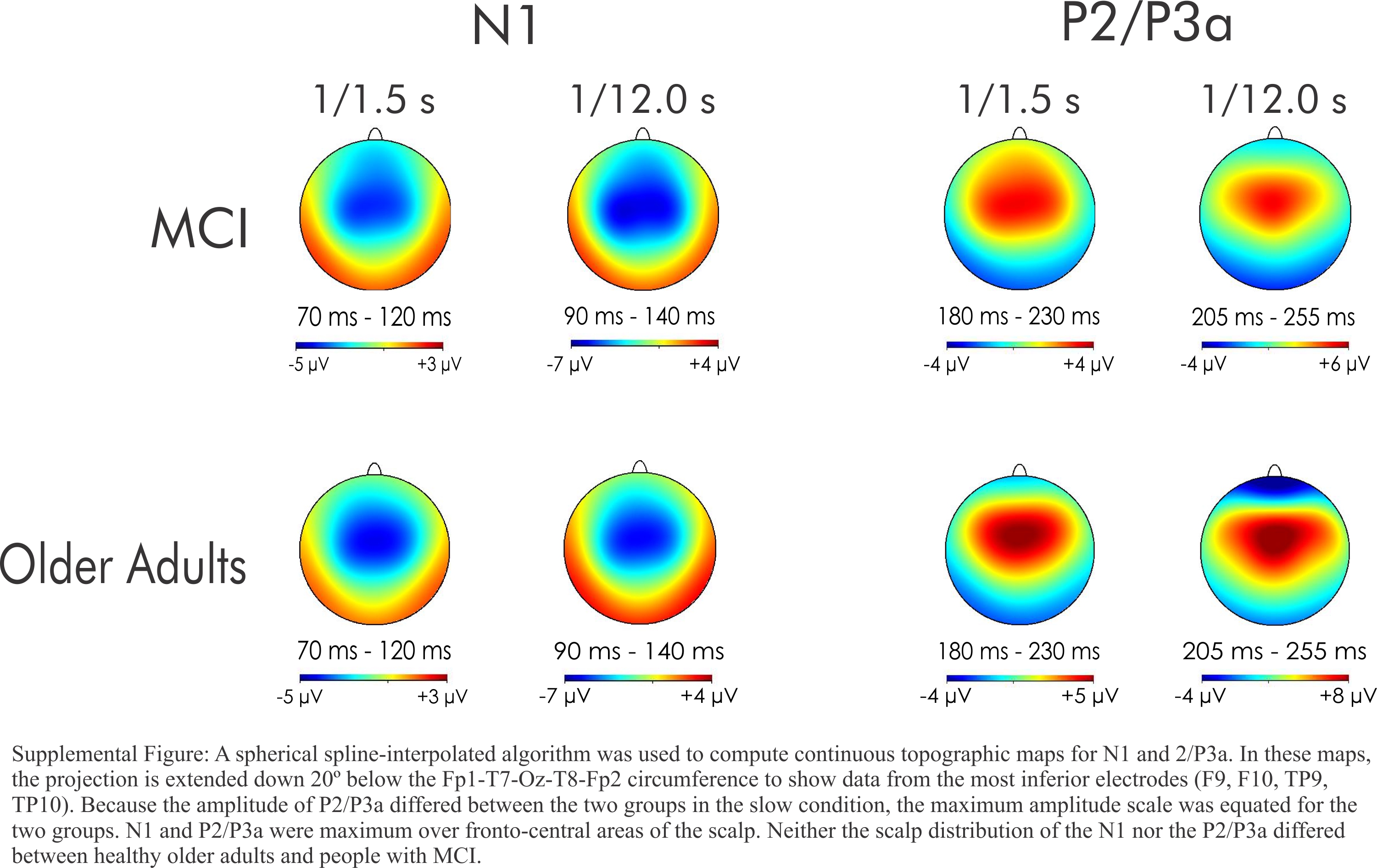

Supplement: Supplementary file 2 [file Image_1.JPEG]
